# High Prevalence and Predictors of Maternal Anemia Risk: Nationwide Midwife-Reported Screening in Indonesia

**DOI:** 10.3390/healthcare14172744

**Published:** 2026-08-28

**Authors:** Ade Jubaedah, Herlyssa Herlyssa, Hetty Astri, Innana Mardhatilla, Fitriani Fitriani, Ery Suhaerlinah, Ray Wagiu Basrowi, Dessy Pratiwi, Nova Lidia Sitorus

**Affiliations:** 1 Indonesian Midwives Association, Johar Baru D13, Central Jakarta, Jakarta 10560, Indonesia; bd.adejubaedah@gmail.com (A.J.); lyssafira3@gmail.com (H.H.); hettyastri@gmail.com (H.A.); nana311@gmail.com (I.M.); bidanfitriani66@gmail.com (F.F.); erysuhaerlinahs2@gmail.com (E.S.); 2 Pelita Ilmu Institute of Health Sciences, Bojongsari 34, Depok 16516, West Java, Indonesia; 3 Health Polytechnic of the Ministry of Health Jakarta III, Melati 2 15, Bekasi 17415, West Java, Indonesia; 4 Department of Community Medicine, Faculty of Medicine, Universitas Indonesia, Salemba Raya 6, Central Jakarta, Jakarta 10430, Indonesia; 5 Indonesia Health Development Center, Percetakan Negara 29, Central Jakarta, Jakarta 10560, Indonesia; pratiwi.dessy@gmail.com; 6 Health Collaborative Center, TB Simatupang 18C, South Jakarta, Jakarta 12430, Indonesia; nova.lidia.sitorus@gmail.com

**Keywords:** maternal anemia, pregnancy, symptom-based screening, digital health, reproductive health, Indonesia, midwife-led care

## Abstract

**Background:** Maternal anemia remains a major public health concern in low- and middle-income countries, including Indonesia, where access to hemoglobin and iron-status measurements remains uneven. Although non-laboratory screening cannot confirm anemia or iron deficiency, digital collection of anemia-related symptoms, clinical signs, nutritional indicators, and reproductive risk factors may support preliminary risk identification in settings with limited diagnostic capacity. This study aimed to describe the nationwide implementation of a digital composite anemia-risk checklist and to examine maternal, reproductive, and regional factors associated with screening-positive status among pregnant women in Indonesia. **Methods:** A retrospective cross-sectional study was conducted using secondary data recorded through the e-Nutri mobile application during routine antenatal care from February to April 2025. The analysis included 205,822 pregnant women recorded across all 34 Indonesian provinces. Screening-positive anemia-related risk was defined operationally as at least one positive response among ten checklist indicators. Associations between maternal age, gestational age, parity, birth spacing, geographic region, and screening-positive status were examined using Poisson regression with robust variance estimation to obtain adjusted prevalence ratios (aPRs) and 95% confidence intervals (CIs). **Results:** Overall, 65.3% of participants met the operational criterion for screening-positive anemia-related risk. Multiparity was associated with a higher prevalence of screening-positive status (aPR 1.20; 95% CI 1.19–1.21), as was a birth interval of less than two years (aPR 1.10; 95% CI 1.09–1.11). Screening-positive status was slightly less prevalent during the second and third trimesters than during the first trimester. Compared with Sulawesi, the adjusted prevalence was higher in Kalimantan and lower in Sumatra and Maluku–Papua. Although 91.6% of participants reported iron supplementation use, the available data did not measure dosage, duration, adherence, or biochemical response. **Conclusions:** Nearly 7 of 10 pregnant women are at risk of anemia. Digital symptom-based screening conducted at a national scale identified widespread maternal anemia risk and consistent associations with cumulative reproductive burden and regional context. This approach potentially offers an alternative complement to laboratory-based surveillance in resource-limited settings.

## 1. Introduction

Anemia during pregnancy, defined by the World Health Organization as hemoglobin levels below 11 g/dL, remains a challenging problem in low- and middle-income source countries (LMICs) like Indonesia. Previous studies [1,2] document that there are about 36–42% of pregnant women in LMICs. No symptoms of early-stage anemia have ever been categorized. However, the disease increases the likelihood of perinatal morbidity and death, postpartum hemorrhage, and maternal fatigue [3,4]. Premature delivery, low birth weight, and worse developmental outcomes are all closely associated with maternal anemia, which has similarly serious repercussions for newborns [5]. Goals 2 and 3 of the Sustainable Development Agenda, which include nutrition and mother–child health, cannot be achieved without resolving this burden [6].

National prevalence of maternal anemia in Indonesia declined from 48.9% in 2018 to 27.7% in 2023 [7,8]; however, these laboratory-confirmed estimates rely exclusively on hemoglobin testing, which remains not widely accessible across regions. Persistent disparities in nutritional intake [9], dietary taboos [10], and antenatal care access [7] sustain anemia vulnerability. Diagnostic infrastructure remains fragmented. Outdated Sahli’s methods are still in use, and validated point-of-care devices such as HemoCue are available in about 88% of public clinics and 50% of private facilities [8,11]. These limitations create significant surveillance blind spots, mainly in remote or resource-constrained settings. For geographic comparison, the 34 provinces were grouped into six broad regions: Sumatra, Java, Bali–Nusa Tenggara, Kalimantan, Sulawesi, and Maluku–Papua. Previous Indonesian evidence has documented geographic disparities in antenatal care utilization [7], while province-level experience from South Kalimantan indicates that routine maternal data collection and service implementation can depend on local training and service capacity [12]. These differences provide a relevant context for regional analysis, although the grouping does not assume that provinces within the same region are homogeneous in population or health-system characteristics.

Laboratory-based assessment remains essential for diagnosing maternal anemia and iron deficiency. Hemoglobin measurement is required to identify anemia, whereas serum ferritin and other iron-status indicators are needed to determine whether anemia is attributable to iron deficiency. However, access to these measurements remains uneven in resource-constrained and geographically dispersed settings [13]. In such contexts, non-laboratory screening cannot confirm iron deficiency but may support preliminary identification of women presenting with anemia-related symptoms, signs, nutritional vulnerabilities, or reproductive risk factors who may require further assessment. Previous symptom- and risk-factor-based screening approaches have generally been evaluated in limited or controlled populations, and evidence regarding their large-scale implementation through routine maternal healthcare systems remains scarce. In particular, it remains unclear whether a digital, midwife-administered composite checklist can be operationalized across Indonesia’s geographically diverse and decentralized health system and whether screening-positive classifications vary according to maternal, reproductive, and regional characteristics. Therefore, this study did not aim to identify iron deficiency or estimate the prevalence of laboratory-confirmed anemia. Instead, it aimed to describe the nationwide implementation of a digital composite anemia-risk screening checklist and to examine maternal, reproductive, and regional factors associated with screening-positive status among pregnant women in Indonesia. We evaluate how reproductive burden, specifically parity and birth spacing, and regional health system variations modify screening outcomes in decentralized settings.

## 2. Materials and Methods

### 2.1. Study Design and Setting

This study employs a cross-sectional design, utilizing secondary data collected from the iron risk screening embedded in the e-Nutri application. The study analyzed existing data without any intervention to assess the relationships among the study variables. e-Nutri is a digital health tool developed to support Indonesian midwives in their routine healthcare practices. The application offers a variety of features aimed at enhancing professional performance, such as access to the latest scientific updates, relevant training modules, expert consultation services, and communication tools to promote collaboration and knowledge sharing among midwives across the country [14,15]. To enhance screening efforts for iron deficiency anemia, the app provides an Iron Calculator as a tool for iron risk screening. The information on iron risk screening from 34 provinces in Indonesia was analyzed during the months of February to April 2025. Adherence to the STROBE (Strengthening the Reporting of Observational Studies in Epidemiology) criteria is maintained throughout the study reporting.

### 2.2. Population and Sampling

**Figure 1:**
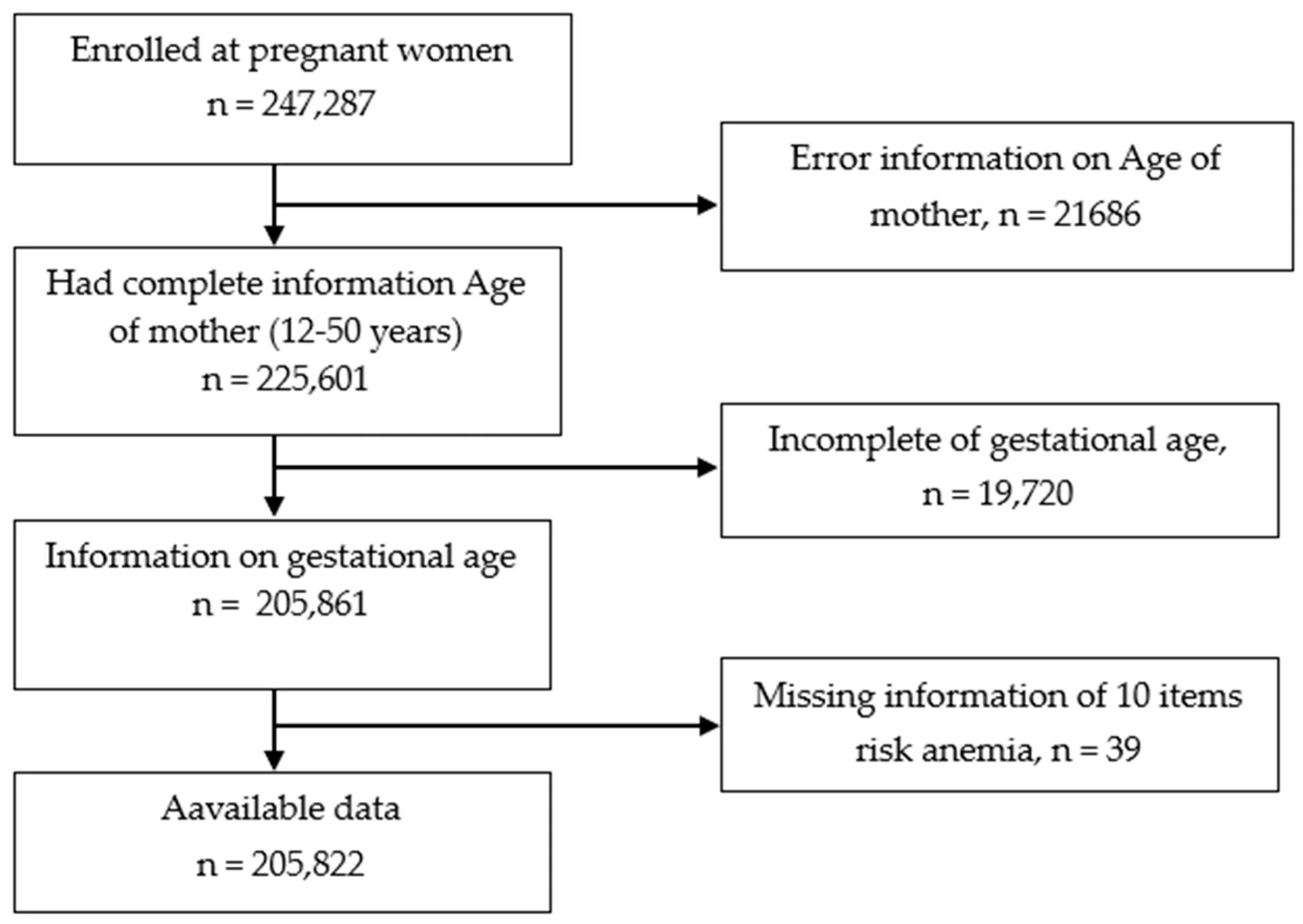
Flow chart showing available data of study participants.

The study population included pregnant women receiving antenatal care services from midwives utilizing the e-Nutri application. Data collection points included Integrated Health Posts (*Posyandu*), independent midwifery clinics (*Praktik Mandiri Bidan*), and home visits/outreach programs. Inclusion criteria were: (1) pregnant women with a recorded gestational age based on the Last Menstrual Period (LMP); (2) women of reproductive age (defined as 12–50 years to capture early marriage and late pregnancy contexts in Indonesia). Exclusion criteria were: (1) records with incomplete or implausible data on key variables. From an initial dataset of 247,287 entries, a total of 205,822 pregnant women met the eligibility criteria and were included in the final analysis (Figure 1).

### 2.3. The e-Nutri Screening Instrument

Maternal anemia risk was assessed using the e-Nutri symptom-based screening checklist. To improve the validity of the collected data, the questionnaire consisted of standardized, clearly worded items embedded in the e-Nutri application, which minimized interviewer variability and promoted consistent data collection across participants. Furthermore, the screening instrument had been previously developed and assessed for reliability and construct-related properties before being incorporated into the application. This instrument is designed as a preliminary triage tool for resource-limited settings and comprises 10 key indicators:

1. Intake of milk for pregnancy preparation: Consumption of milk or milk-based fortified products (e.g., containing calcium, iron, or folic acid) by women before or during preparation for pregnancy, either regularly or irregularly.
2. History of anemia diagnosis: A self-reported or medically documented history of being diagnosed with anemia by a healthcare professional based on low hemoglobin levels.
3. Multiple gestation: A pregnancy in which more than one fetus is present, such as twin or higher-order multiple pregnancies.
4. History of morning sickness/nausea: A history of experiencing nausea and/or vomiting, particularly during the early stages of pregnancy.
5. Low intake of animal protein: Getting less protein than the required daily allowance from foods derived from animals, such as meat, fish, eggs, and dairy.
6. Low intake of fortified milk: Infrequent or insufficient consumption of milk fortified with essential micronutrients such as iron, folic acid, calcium, or vitamins, compared to recommended intake levels.
7. Irregular consumption of Iron-Folic Acid (IFA) supplements: Inconsistent intake of iron–folic acid tablets, including missed doses or consumption that does not follow the recommended dosage or frequency.
8. Chronic fatigue/lethargy: Persistent feelings of tiredness, weakness, or lack of energy lasting over a prolonged period and not relieved by adequate rest.
9. Physical signs of pallor (facial/conjunctival): Observable paleness of the face or conjunctiva of the eyes on physical examination, commonly associated with anemia.
10. Nail discoloration/brittleness: Changes in nail color and/or increased nail fragility, including brittle, thin, or easily broken nails, which may indicate iron deficiency or other nutritional deficiencies.

Each indicator represents a biologically plausible pathway contributing to anemia risk, with established mechanistic links to impaired hemoglobin production or increased iron requirements, which are reflected through clinical symptoms. Accordingly, a pregnant woman is classified as “High Risk” if she screened positive for at least one of these indicators, as even a single positive finding may indicate significant physiological vulnerability to anemia or early manifestations of iron deficiency. Considering the heightened nutritional demands of pregnancy and the preventive intent of symptom-based screening, applying a ≥1 indicator threshold improves sensitivity and facilitates timely identification and intervention, rather than delaying action until multiple risk factors coexist.

*Note on Validation:* It is important to state that this e-Nutri tool is a symptom-based screening algorithm and has not yet been diagnostically validated against serum hemoglobin or ferritin levels. Therefore, the outcome is defined as “Anemia Risk” rather than clinical anemia. Previous assessments demonstrated acceptable internal consistency (Cronbach’s α = 0.728), while Pearson correlation coefficients ranged from 0.311 to 0.538, supporting the questionnaire’s reliability and construct-related assessment. The questionnaire was subsequently incorporated into the e-Nutri digital application, which has been implemented for use by Indonesian midwives.

### 2.4. Variables and Operational Definitions

- Outcome Variable: Maternal Anemia Risk (Binary: High Risk vs. Low Risk), generated automatically by the Iron Calculator algorithm within the e-Nutri application.
- Maternal Age: Categorized into <26 years and ≥26 years, based on birth date. Data were collected directly from mothers through face-to-face questioning. We used 26 years as the age cutoff because the age variable was not normally distributed. Therefore, the median age (26 years) was used as the cutoff to categorize participants into two groups.
- Gestational Age: Gathered by directly asking at screening regarding the first day of the mother’s last menstrual period, then classified based on standard trimester definitions:
  - First Trimester: 0–13 weeks.
  - Second Trimester: 14–26 weeks.
  - Third Trimester: 27–40 weeks.
- Reproductive History: Directly collected from mothers through face-to-face asking:
  - Parity: The number of previous live births at screening and categorized as: Nulliparous (0 previous births), Primiparous (1 previous birth), and Multiparous (≥3 previous births).
  - Birth Spacing: Interval between the most recent previous birth and the current pregnancy based on the age of the children at screening. Categorized as First pregnancy (not applicable), <2 years (short interval), and ≥2 years.
- Region: Generated automatically from midwives’ domicile, categorized geographically into Sumatera, Java, Nusa Tenggara & Bali, Kalimantan, Sulawesi, Maluku, and Papua.

### 2.5. Statistical Analysis

The statistical package SPSS, version 26 (IBM Corp., Armonk, NY, USA), was used for dataset cleansing and analysis. To summarize demographic information, descriptive statistics were used. Categorical variables were presented as frequencies and percentages. To estimate the association between independent variables and anemia risk, we calculated Prevalence Ratios (PR) rather than Odds Ratios, as the outcome was common (>10%). Univariable analysis was performed for each predictor. For multivariable analysis, to avoid the “Table 2 Fallacy,” we did not enter all variables into a single model. Instead, we utilized a conceptual framework based on Directed Acyclic Graphs (DAGs) to identify specific potential confounders for each exposure–outcome relationship. We employed Poisson Regression with Robust Variance Estimation (generalized linear models with log link and robust error variances) to obtain adjusted Prevalence Ratios (aPR) and 95% Confidence Intervals (CIs). This method provides more accurate estimates for cross-sectional binary outcomes compared to Cox regression or logistic regression. A *p*-value < 0.05 was considered statistically significant.

### 2.6. Ethical Considerations

The Research Institute of Universitas Yarsi granted ethical approval for this work (No: 264/KEP-UY/EA.10/VIII/2025). The ethics committee decided not to seek formal informed consent as this research used secondary data that was already collected but had personal information removed. This research followed all applicable laws and guidelines on the protection of personal health information in Indonesia, as well as the Declaration of Helsinki.

## 3. Results

### 3.1. Characteristics of Pregnant Women

A total of 8426 midwives providing routine maternal and child healthcare services participated in this study. The study included 205,822 participants from 34 provinces across Indonesia. The largest proportions of participants were from East Java (14.9%), South Sulawesi (11.9%), South Sumatra (8.3%), Central Java (8.0%), Gorontalo (7.5%), and Bengkulu (7.2%). In contrast, the smallest proportions were from Papua, East Nusa Tenggara, North Kalimantan, Maluku, and North Maluku, each contributing less than 0.1% of the total sample.

Table 1 shows data from 205,822 pregnant women with a mean age of 27.38 years (SD = 5.36). Most women were <26 years old (54.8%) and in their second trimester of pregnancy (42.0%). A substantial 69.1% of the women in the study were nulliparous, indicating that a large proportion were experiencing their first pregnancy. The prevalence risk of anemia among the mothers was 65.3%. Most participants were from Sumatra (42.3%). Only 0.5% came from Maluku and Papua.

### 3.2. Distribution of Iron Deficiency Anemia Screening Parameters

The mean total positive-item score was 1.88 (SD = 2.05), while the median score was 1.00, indicating that at least half of the participants reported only one or fewer positive items. Scores ranged from 0 to 10, demonstrating substantial variability in the number of positive responses across participants. Among participants screened, 71.5% of women reported drinking milk for pregnancy preparation, while 19.0% had an anemia history, 15.3% had multiple gestation, 20.0% had fatigue, 14.9% had pallor, and 13.7% had brittle nails (Table 2).

### 3.3. Factors Associated with Anemia Risk Among Pregnant Women

Several predictor factors were shown to be associated with anemia in Table 3 and Table 4. Similar patterns were seen in both the bivariate and multivariate studies. Compared to younger mothers, moms aged 26 and over had a 2% decreased incidence of anemia (aPR 0.98; 95% CI: 0.97–0.99). The risk of anemia was also substantially related to the third trimester of pregnancy. Compared with the first trimester, the second and third trimesters were associated with a 4% and 2% decrease, respectively, in the risk of anemia in pregnant women. There was a favorable correlation between parity and anemia. The risk of anemia was 20% greater in multiparous women (≥3 prior births) compared to nulliparous women (aPR 1.20; 95% CI: 1.19–1.21) and 7% higher in primiparous women (aPR 1.07; 95% CI: 1.06–1.08). Similarly, the risk was 10% greater for moms whose births were fewer than two years apart compared to those whose births were more than two years apart (aPR 1.10; 95% CI: 1.09–1.11). Anemia risk was reduced in women from Sumatra (aPR 0.92; 95% CI 0.91–0.93) and Maluku-Papua (aPR 0.91; 95% CI 0.87–0.95) compared to Sulawesi (reference), but somewhat higher in Kalimantan (aPR 1.03; 95% CI 1.01–1.05). The link between maternal age and the likelihood of anemia differed between regions. Table 5 shows that, in contrast to other locations, a slightly elevated risk was associated with older maternal age (≥26 years) in Sulawesi (aPR 1.01; 95% CI 1.01–1.02).

Table 5 presents the association between the risk of anemia among maternal populations across regions in Indonesia. In Sulawesi, maternal age ≥26 years had a higher risk of anemia. In contrast, mothers in Sumatra, Java, Nusa Tenggara and Bali, Kalimantan, and Maluku–Papua had a lower risk of anemia in the same age group. Anemia risk was lower in the second and third trimesters in all regions. The number of pregnancies was consistently associated with increased anemia risk across all regions, where a higher parity corresponded to a greater risk of anemia among mothers. Finally, all regions showed consistent findings regarding birth spacing: mothers with less than two years of birth interval had a higher risk of anemia compared to those with more than two years.

## 4. Discussion

### 4.1. Interpretation of Symptom-Based Anemia-Risk Classification

This study applied a 10-item digital composite checklist to 205,822 pregnant women recorded through the e-Nutri platform across Indonesia. The checklist included anemia-related symptoms, observable clinical signs, dietary and supplementation-related indicators, pregnancy-related conditions, and previous anemia history [16,17,18,19,20]. The platform enabled standardized large-scale collection of these screening indicators within Indonesia’s decentralized maternal health system.

The tool classified 65.3% of participants as screening positive for anemia-related risk. This proportion is not directly comparable with national laboratory-confirmed anemia estimates of 27.7% in 2023 and 48.9% in 2018 [5,9,13], because the measures represent different constructs. National estimates were based on hemoglobin measurements, whereas the e-Nutri outcome was based on the composite checklist.

The current study did not compare e-Nutri classifications with laboratory findings in the same participants and therefore cannot determine concordance, sensitivity, specificity, predictive values, or diagnostic accuracy. The ≥1-indicator threshold produced a broad classification, but its diagnostic performance has not been established.

Several features of the checklist may contribute to the high screening-positive proportion. The threshold requires only one positive response, and some indicators are not specific to current anemia. Pallor and previous anemia history may indicate the need for further assessment, whereas fatigue and nausea may overlap with normal pregnancy physiology. Dietary and supplementation-related items indicate potential vulnerability but do not confirm current anemia or iron deficiency [21,22].

Accordingly, digital collection through e-Nutri should be interpreted as an operational approach for documenting screening-positive anemia-related risk rather than as a validated diagnostic or surveillance tool. The platform may be relevant in settings where point-of-care hemoglobin testing is not universally available [6,23]. Nevertheless, the present findings do not establish that e-Nutri can accurately identify anemia, detect early disease, or function as a first-line clinical triage instrument. Prospective studies should compare the checklist with hemoglobin and serum ferritin and examine whether the current threshold or alternative scoring approaches provide clinically useful discrimination.

### 4.2. Reproductive Factors Associated with Screening-Positive Anemia-Related

Multiparity (≥3 previous births) and short interpregnancy intervals (<2 years) demonstrated strong associations with elevated anemia risk (aPR 1.20 and 1.10, respectively). These patterns reinforce the maternal depletion hypothesis, which posits that repeated pregnancies without adequate recovery erode micronutrient reserves [5,24].

The adjusted analyses showed that the reproductive associations remained evident after accounting for the included covariates. Older maternal age (≥26 years) was associated with slightly lower screening-positive prevalence in most regions, while Sulawesi showed a small positive association (aPR 1.01; 95% CI 1.01–1.02). These region-stratified estimates are descriptive and should not be interpreted as evidence of effect modification because formal interaction testing was not performed.

Possible explanations related to education, health literacy, healthcare access, economic resources, and healthcare utilization have been discussed in previous literature [25], but these factors were not directly measured in the present study. They should therefore consider contextual hypotheses rather than mechanisms demonstrated by the data.

The different age pattern observed in Sulawesi requires confirmation in independent samples with adequate regional representation and direct measurement of relevant socioeconomic and healthcare factors. Reproductive history may be considered in future risk-classification models, but the present findings do not establish these variables as diagnostic predictors of laboratory-confirmed anemia. Future models should be developed and validated against biochemical outcomes before parity, birth spacing, or maternal age is used to guide supplementation, referral, or confirmatory testing.

### 4.3. Regional and Province-Level Variation in Screening-Positive Status

Regional analysis showed variation in screening-positive status. Compared with Sulawesi, Kalimantan had a slightly higher adjusted prevalence ratio (aPR 1.03), while Sumatra and Maluku–Papua had lower ratios (aPR 0.92 and 0.91, respectively). These estimates describe differences within the e-Nutri dataset and not population-level differences in laboratory-confirmed anemia or iron deficiency.

Potential contextual explanations include differences in screening practices, diet, urbanization, sanitation, healthcare access, and platform implementation [1,6,21,26,27,28]. Previous Indonesian studies have documented geographic variation in antenatal care utilization [7] and highlighted the importance of local training and implementation capacity for strengthening routine antenatal care data collection [12]. Preventive measures for maternal anemia, including iron–folic acid supplementation, nutritional assessment and counseling, antenatal follow-up, and access to diagnostic assessment when indicated, are broadly applicable but may require different operational emphasis across regional service contexts [22]. However, these factors were not directly measured in the present study and therefore should be considered contextual considerations rather than established explanations for the observed regional patterns.

Participant numbers varied substantially across regions, from 955 women in Maluku–Papua to 87,083 in Sumatra, while the screening-positive proportion ranged from 62.61% in Sumatra to 72.12% in Kalimantan. The estimate for Maluku–Papua should be interpreted with particular caution given this region contributed only 0.5% of the total sample. In addition, the use of routine platform data rather than regionally representative probability sampling means that regional estimates may be influenced by unequal sample sizes, selective platform coverage, participant characteristics, and differences in screening or reporting practices. Regional grouping also provides less geographic detail than province-level analysis. Therefore, representative province-level sampling combined with laboratory confirmation would be required before geographic differences could be interpreted as reflecting differences in the underlying burden of maternal anemia or iron deficiency.

### 4.4. Reported Iron Supplementation and the Provincial Fe Tablet Program

Although 91.6% of participants reported iron supplementation, 65.3% met the screening-positive criterion. These findings should not be interpreted as evidence that iron supplementation or the national Fe tablet program was ineffective, because the dataset did not measure the number of tablets received or consumed, timing, duration, regularity of intake, adherence, or biochemical response.

Indonesian literature documents persistent barriers, including perceived side effects, concerns about delivering “large babies,” and inadequate counseling [29,30]. Women may discontinue tablets because of gastric discomfort or misinformation within their families [9,30,31]. The Indonesian literature provides relevant context regarding counseling, adverse effects, family beliefs, and adherence [9,29,30,31], but these factors were not measured in e-Nutri and cannot be assumed to explain the present findings.

Other possible contributors include late or inconsistent supplementation, dietary factors, infection, inflammation, blood loss, or pre-existing iron deficiency; however, none of these mechanisms could be evaluated using the available dataset. The present study therefore cannot determine whether the high screening-positive proportion reflects supplementation adherence, program effectiveness, non-iron causes of the recorded symptoms and signs, or the broad ≥1-indicator threshold. Individual-level data linking tablet receipt and actual consumption with dietary and biochemical measurements would be required to evaluate these possibilities.

### 4.5. Strengths and Limitations

The study’s large sample size (*n* = 205,822) provides robust estimates for screening-positive proportions and main-effect associations, enabling the detection of even modest differences across demographic and reproductive subgroups. The inclusion of screening records from all 34 Indonesian provinces also demonstrates the operational reach of a digital, midwife-led data-collection platform within a geographically dispersed maternal healthcare system.

The main limitation is that the e-Nutri digital composite checklist has not been diagnostically validated against hemoglobin or ferritin in this cohort. The checklist combines symptoms, observable clinical signs, dietary and supplementation-related indicators, pregnancy-related conditions, and previous anemia history. Some items may overlap with normal pregnancy physiology or reflect vulnerability rather than current disease, while self-reported or observer-assessed items may be affected by recall, social desirability, differences in interpretation, and inter-observer variation. Therefore, the study cannot determine diagnostic accuracy, sensitivity, specificity, predictive values, or the optimal classification threshold.

Regional estimates should also be interpreted cautiously because participant distribution was uneven, particularly in Maluku–Papua (*n* = 955; 0.5%). Region-stratified associations, including the different age pattern observed in Sulawesi, require replication and formal interaction testing before they can support geographically targeted interpretations. Similarly, associations with multiparity and short birth intervals were observed across all six regions, but they remain associations with screening-positive status rather than predictors of laboratory-confirmed anemia or iron deficiency.

The study used routine platform data rather than probability-based national sampling. Participation depended on e-Nutri use by participating midwives and the availability of complete screening records; therefore, the findings may not represent all pregnant women in Indonesia, and regional or province-level estimates should not be interpreted as population prevalence estimates. The cross-sectional design also prevents conclusions regarding temporality or causality, and residual confounding is possible because dietary intake, socioeconomic conditions, educational attainment, infection, inflammation, healthcare utilization, and household food security were not measured.

Any comparison with provincial Fe tablet program indicators is ecological because program-level data were not linked to individual e-Nutri records; such comparisons should therefore remain descriptive. Despite these limitations, the study demonstrates the feasibility of collecting standardized anemia-related indicators digitally at a large operational scale. Clinical triage, population surveillance, supplementation decisions, or resource allocation would require prospective biochemical validation.

### 4.6. Looking Ahead: Toward Intergenerational Anemia Surveillance

Future research should prioritize prospective validation of the e-Nutri checklist against hemoglobin, serum ferritin, and other clinically appropriate iron-status indicators. Validation studies should assess the contribution of individual items, examine inter-observer reliability, report the distribution of total positive-item scores, and compare alternative scoring thresholds. External validation across provinces, healthcare settings, and participant populations will also be required to determine whether model performance remains stable in different implementation contexts. Only after these steps have been completed should the potential clinical or public-health applications of the checklist be evaluated. These applications may include supporting decisions regarding confirmatory assessment, identifying groups for further evaluation, or contributing to routine health-information systems. Adaptation to postpartum women or children would require separate instrument development and population-specific validation because symptoms, clinical indicators, reference standards, and causes of anemia differ across pregnancy, the postpartum period, infancy, and childhood. Therefore, the current pregnancy checklist should not be assumed to have valid intergenerational, postpartum, or pediatric surveillance utility without further evidence.

## 5. Conclusions

Nearly seven in ten participants met the operational criterion for screening-positive anemia-related risk. Multiparity and a birth interval of less than two years were associated with a higher prevalence of screening-positive status, while screening-positive status was slightly less prevalent in the second and third trimesters than in the first trimester. These findings describe maternal and reproductive factors associated with the operational checklist classification and should not be interpreted as the prevalence of, or predictors for, laboratory-confirmed anemia or iron deficiency.

Using routine data from a midwife-led digital platform across all 34 Indonesian provinces, this study demonstrates the feasibility of collecting standardized anemia-related indicators at substantial operational scale. Because the e-Nutri checklist has not been diagnostically validated against hemoglobin or serum ferritin, the present study does not establish diagnostic accuracy, surveillance accuracy, or clinical triage utility. Prospective biochemical validation, including evaluation of alternative scoring thresholds across different provinces and healthcare settings, is required before the checklist can be used for clinical triage or population-level anemia surveillance.

## Figures and Tables

**Table 1:** Characteristics of pregnant women (*n* = 205,822).

Variable	*n* (%)	Mean ± SD	Median (IQR)
**Age of mother (years)**		27.38 ± 5.36	26.00 (24.00–31.00)
<26	112,858 (54.8)		
≥26	92,964 (45.2)		
**Gestational age**		5.07 ± 2.30	5.00 (3.00–7.00)
First trimester	59,364 (28.8)		
Second trimester	86,443 (42.0)		
Third trimester	60,015 (29.2)		
**Number of pregnancies**		1.00 ± 2.41	1.00 (1.00–2.00)
I	142,295 (69.2)		
II	37,756 (18.3)		
>III	25,771 (12.5)		
**Reproductive History**			
** Birth Spacing**		1.00 ± 2.41	0.00 (0.00–1.08)
First-time pregnant	142,295 (69.1)		
less than 2 years	18,107 (8.8)		
more than 2 years	45,420 (22.1)		
**Parity**		0.50 ± 0.91	0.00 (0–1.00)
Nulliparous	142,295 (69.1)		
Primiparous	37,756 (18.3)		
Multiparous	25,771 (12.5)		
**Anemia risk of maternal**			
No	71,420 (34.7)		
Yes	134,402 (65.3)		
**Region**			
Sumatera	87,083 (42.3)		
Java	59,793 (29.1)		
Nusa Tenggara & Bali	4274 (2.1)		
Kalimantan	4864 (2.4)		
Sulawesi	48,853 (23.7)		
Maluku & Papua	955 (0.5)		

**Table 2:** Ten-item risk of anemic pregnant women (*n* = 205,822).

Items	*n* (%)
**Intake of milk for pregnancy preparation**	
Yes	147,067 (71.5)
No	58,755 (28.5)
**History of anemia**	
No	166,789 (81.0)
Yes	39,033 (19.0)
**Multiple gestation**	
No	174,282 (84.7)
Yes	31,540 (15.3)
**Morning sickness**	
No	129,320 (62.8)
Yes	76,502 (37.2)
**Intake of animal-source foods**	
Yes	165,022 (80.2)
No	40,800 (19.8)
**Intake of milk for pregnancy**	
Yes	182,343 (88.6)
No	23,479 (11.4)
**Intake iron supplementation**	
Yes	188,583 (91.6)
No	17,239 (8.4)
**Fatigue symptoms**	
No	164,698 (80.0)
Yes	41,124 (20.0)
**Physical signs of pallor**	
No	175,179 (85.1)
Yes	30,643 (14.9)
**Brittle nails**	
No	177,633 (86.3)
Yes	28,189 (13.7)

**Table 3:** Bivariate analysis of risk factors associated with anemia in pregnant women (*n* = 205,822).

Variable	Anemia Risk of Pregnant Women				Unadjusted PR (95% CI)	*p*-Value
	No		Yes			
	*n*	%	*n*	%		
**Age of mother (years)**						
<26	38,313	33.9	74,545	66.05	1.00	
≥26	33,107	35.6	59,857	64.39	1.03 (1.02–1.03)	<0.001 *
**Gestational age**						
First trimester	20,031	33.7	39,333	66.26	1.00	
Second trimester	30,840	35.7	55,603	64.32	0.97 (0.96–0.98)	<0.001 *
Third trimester	20,549	34.2	39,466	65.76	0.99 (0.98–1.00)	0.070
**Reproductive History**						
** Birth Spacing**						
more than 2 years	13,867	30.5	31,553	69.47	1.00	
First-time pregnant	53,359	37.5	88,936	62.50	0.90 (0.89–0.91)	<0.001 *
less than 2 years	4194	23.2	13,913	76.84	1.12 (1.09–1.12)	<0.001 *
** Parity**						
Nulliparous	53,359	37.5	88,936	62.50	1.00	
Primiparous	11,947	31.6	25,809	68.36	1.09 (1.08–1.10)	<0.001 *
Multiparous	6114	23.7	19,657	76.28	1.22 (1.20–1.23)	<0.001 *
**Region**						
Sulawesi	15,553	31.8	33,300	68.16	1.00	
Sumatera	32,559	37.4	54,524	62.61	0.92 (0.91–0.93)	<0.001 *
Jawa	20,370	34.1	39,423	65.93	0.97 (0.96–0.98)	<0.001 *
Nusa Tenggara & Bali	1235	28.9	3039	71.10	1.04 (1.02–1.06)	<0.001 *
Kalimantan	1356	27.9	3508	72.12	1.06 (1.04–1.08)	<0.001 *
Maluku & Papua	347	36.3	608	63.66	0.93 (0.89–0.98)	0.006

Note: * significance 5%.

**Table 4:** Multifactor Poisson regression with robust variance estimation of risk factors associated with anemia in pregnant women (*n* = 205,822).

Variables	*p*-Value	Adjusted PR	95% CI	
			Lower	Upper
**Age of mother (years)**				
<26	1.00			
≥26	<0.001 *	0.98	0.97	0.99
**Gestational age**				
First trimester		1.00		
Second trimester	<0.001 *	0.96	0.96	0.97
Third trimester	0.003 *	0.98	0.97	0.99
**Reproductive History**				
** Birth Spacing**				
more than 2 years		1.00		
less than 2 years	<0.001 *	1.10	1.09	1.11
** Parity**				
Nulliparous		1.00		
Primiparous	<0.001 *	1.07	1.06	1.08
Multiparous	<0.001 *	1.20	1.19	1.21
**Region**				
Sulawesi		1.00		
Sumatera	<0.001 *	0.92	0.91	0.93
Java	<0.001 *	0.98	0.97	1.00
Nusa Tenggara & Bali	0.029	1.02	1.00	1.04
Kalimantan	0.002	1.03	1.01	1.05
Maluku & Papua	<0.001 *	0.91	0.87	0.95

Note: * significance 5%.

**Table 5:** Multi-factor Poisson regression with robust variance estimation of risk factors associated with anemia in pregnant women across regions in Indonesia (*n* = 205,822).

Variable	Sulawesi	Sumatera	Java	Nusa Tenggara and Bali	Kalimantan	Maluku and Papua
**Age of mother (years)**						
<26	1.00	1.00	1.00	1.00	1.00	1.00
≥26	1.01 (1.01–1.02) *	0.95 (0.94–0.96) *	0.97 (0.96–1.00) *	0.98 (0.98–1.00) *	0.98 (0.98–0.99) *	0.98 (0.97–0.99) *
**Gestational age**						
First trimester	1.00	1.00	1.00	1.00	1.00	1.00
Second trimester	0.95 (0.94–0.96) *	0.97 (0.96–0.98) *	0.97 (0.96–0.98) *	0.96 (0.95–0.96) *	0.96 (0.95–0.97) *	0.96(0.95–0.97) *
Third trimester	0.97 (0.96–0.97) *	0.97 (0.96–0.98) *	1.01 (1.00–1.02) *	0.97 (0.96–0.98) *	0.98 (0.97–0.98) *	0.97 (0.97–0.98) *
**Reproductive History**						
**Birth Spacing**						
more than 2 years	1.00	1.00	1.00	1.00	1.00	1.00
less than 2 years	1.08 (1.07–1.10) *	1.12 (1.10–1.13) *	1.12 (1.10–1.13) *	1.10 (1.08–1.11) *	1.10 (1.09–1.12) *	1.10 (1.09–1.11) *
**Parity**						
Nulliparous	1.00	1.00	1.00	1.00	1.00	1.00
Primiparous	1.07 (1.06–1.08) *	1.09 (1.08–1.10) *	1.04 (1.04–1.06) *	1.08 (1.07–1.09) *	1.07 (1.06–1.08) *	1.76 (1.45–2.15) *
Multiparous	1.19 (1.17–1.20) *	1.22 (1.21–1.23) *	1.19 (1.18–1.21) *	1.20 (1.19–1.21) *	1.20 (1.19–1.21) *	1.77 (1.46–2.16) *

Note: * significance 5% (13).

## Data Availability

The data are available from the corresponding author upon reasonable request due to privacy restrictions.

## Abbreviations

The following abbreviations are used in this manuscript:

aPR	Adjusted Prevalence Ratio
ANC	Antenatal Care
CI	Confidence Interval
DAG	Directed Acyclic Graph
IFA	Iron-Folic Acid
LMIC	Low- and Middle-Income Country
LMP	Last Menstrual Period
PR	Prevalence Ratio
SDG	Sustainable Development Goal
SPSS	Statistical Package for the Social Sciences
STROBE	Strengthening the Reporting of Observational Studies in Epidemiology
WHO	World Health Organization
